# Supplementary material for: Overexpression of Abiotic Stress-Responsive SsCor413-1 Gene Enhances Salt and Drought Tolerance in Sugarcane (Saccharum spp. Hybrid)
Source: Int J Mol Sci. 2025 Oct 10;26(20):9868. doi: 10.3390/ijms26209868 (PMC12563639; doi:10.3390/ijms26209868)
Supplement: Supplementary file 1 [file ijms-26-09868-s001.zip › ijms-3863133-supplementary.pdf]

International Journal of
Molecular Sciences

MDPI

Table S1. Primers used for expression studies of abiotic stress responsive genes

S.No	Name	Sequence (5′-3′)
1	cor413-1F	AGCTTCCTGGTTCCATCATC
	cor413-1R	CATCCAATCGCAAGGCATATC
2	LEA3-rtFP	CAAGGACGCGGTGATGAA
	LEA3- rtRP	TGATCTCTGGTGATGGTGGA
3	POT5-rtFP	GATTTCCCTGTTCCTCCTTACC
	POT5-rtRP	GCACCGAAATAGGAGGAGTTAG
4	DREB2-rtFP	AAGCTGCCCATGCATACGATGA
	DREB2-rtRP	ACACTAGATGCCAGCAACGAAAGC
5	CAL-rtFP	CCGTCGCTGTCCAAGAAG
	CAL-rtRP	ATCTCGTCGAGGGTGATCT
6	FAD-rtFP	TGCTCGCATTCCCGTTATAC
	FAD-rtRP	CGTCGATGTTATGATGTCCTTCT
7	TIP2-rtFP	CCATCAAGGCCTACGTGTC
	TIP2-rtRP	CGCCGTCATTGGTCAGTT
8	DIR-rtFP	TTCAAACCAAGCGAGAGGAA
	DIR-rtRP	CTGACCATCTTGTGGGATGTAA
9	SRP - rtFP	CCAAGGTGGTAATCCTCAAGAC
	SRP - rtRP	TCCCAGCAGTGTCAACATAAG

Porteresia Ubiquitin Promoter Deletion (PD2) Sequence (882bp)

ACTATCACCCTCGAGGTGGAGAGCAGCGACACCATCGACAATGTCAAGGCAAAGATCCAG-
GACAAGGCTATAGGGCTCGAGCGGCCGCCCGGGCAGGTATTTAAGTCTCTTTTTATTTTTCTTCTCTTATATTATTATTGTCG
CGACTTGAGATTTCACTATGAGTGCCTTTACAACTCTTCATTAGATATATCTAAAC-
CATCTTAAACGATATTGGATAATTTTTTTAATTGATATTATTTCTAATCTATTATGTATATTATCATTTTATACTTGGTCTATTT
TTAGAAAATCATAAATCCAACCTAATATATAATTATTCTTAAC-
TACTTCTCTCGTGGCCAAACCAAATGTAAATGCACCATCTTGTACAGGTAAGGTCACAGATTAAGGTCATAGGAGGGTTTA
TATTATTTTGAGGAATTTTATCATGGATTCATCCTAATCACTTTGTCTAGACAGTACTGTG-
TATTTGTGTATTTATGATGGATATATTACTATAACTCAGGAGTATTTTCACAAGAAAATGCTCATATACATTTATTTTTATTA
ACACACATGTATACTTATCCCAATAAATAATTTTTAAGATATTGAATCGATATATCTA-
GAAGTCTGTATAAACATATAGTAGAAAAAAATGTTATAGACATGTACCAAATACGAAATTGTTCATGTTGTTTCATGTGTA
ATCCTAATACACAAAACATCCTCTTTTTTACGTTCCCAGCACCACTAAAAAACAAAC-
TATTTTCTTTTAAGAAAAGGAAAACAAATTCTAGTATAGGCGGAAAGCAGCGGGCCACCCACGTCAGCGAATACGGAGG
AGCGGTTTGACGGCGTGAGCCAAACCTAACGGCGACCAACCAACAAACAAAAAGA
